# Fabrication and Characterisation of the Graphene Ring Micro Electrode (GRiME) with an Integrated, Concentric Ag/AgCl Reference Electrode

**DOI:** 10.3390/s130303635

**Published:** 2013-03-14

**Authors:** James W. Dickinson, Michael Bromley, Fabrice P. L. Andrieux, Colin Boxall

**Affiliations:** Engineering Department, Lancaster University, Lancaster LA1 4YR, UK; E-Mails: j.dickinson2@lancaster.ac.uk (J.W.D.); m.bromley@lancaster.ac.uk (M.B.); F.Andrieux@lancaster.ac.uk (F.P.L.A.)

**Keywords:** graphene electrode, ring electrode, graphene electrochemistry, nano

## Abstract

We report the fabrication and characterisation of the first graphene ring micro electrodes with the addition of a miniature concentric Ag/AgCl reference electrode. The graphene ring electrode is formed by dip coating fibre optics with graphene produced by a modified Hummers method. The reference electrode is formed using an established photocatalytically initiated electroless deposition (PIED) plating method. The performance of the so-formed graphene ring micro electrodes (GRiMEs) and associated reference electrode is studied using the probe redox system ferricyanide and electrode thicknesses assessed using established electrochemical methods. Using 220 μm diameter fibre optics, a ∼15 nm thick graphene ring electrode is obtained corresponding to an inner to outer radius ratio of >0.999, so allowing for use of extant analytical descriptions of very thin ring microelectrodes in data analysis. GRiMEs are highly reliable (current response invariant over >3,000 scans), with the concentric reference electrode showing comparable stability (current response invariant over >300 scans). Furthermore the micro-ring design allows for efficient use of electrochemically active graphene edge sites and the associated nA scale currents obtained neatly obviate issues relating to the high resistivity of undoped graphene. Thus, the use of graphene in ring microelectrodes improves the reliability of existing micro-electrode designs and expands the range of use of graphene-based electrochemical devices.

## 1. Introduction

Micro- and nano-electrochemistry can be used in the study of elementary electrochemical processes such as reactant mass transport and electron transfer, and of fundamental structures such as the electrical double layer [1–4]. Insights derived from these studies find use in technologies such as energy generation and storage (fuel cells [5], supercapacitors [6]), metrology (electroanalysis [7], scanning probe microscopy [8,9]), high spatial/temporal resolution biological studies (inter/intra cellular measurement [10]) for in situ applications such as point-of-care devices in clinical analysis, molecular electronics (single molecule electrochemistry [11]) and environmental monitoring, the latter including analysis of toxic and radioactive ions [12].

Developments within the fields of micro/nanoelectrochemistry are driven by the accessing of ever smaller electrode dimensions. This in turn depends crucially upon reliable fabrication of geometrically well-defined electrodes with sizes in the sub μm range [9]. 2D materials offer the possibility of a new paradigm in the design of such electrodes and with material thickness being exploited to produce electrodes with characteristic dimensions on the nm/sub-nm scale. Graphene is the most attractive material in this context because of its layer thickness of 0.334 nm [13], high conductivity and the generally high electrochemical activity of carbon based materials [14]. In light of the latter property, the electrochemistry of C-based materials has been widely studied predominantly for sensor and energy storage applications [15,16]. However, the electrochemistry of graphene specifically has hitherto been less widely studied, those reports that do exist being concerned with devices that exhibit a mixed edge/basal plane response [17].

Additionally, widespread adoption of micro and especially nanosensor technology is currently constrained by the quality and expense of fabrication of these devices on a large scale. While miniaturisation of electrochemical sensors has advanced dramatically during the last two decades the development of a reliable miniature reference electrode has thus far hindered further reductions in scale of modular electrochemical sensors, comprising of working, reference and counter electrodes. The reference electrode is an integral part of any electrochemical measurement and previous efforts in miniaturisation and simplification of electrochemical measuring systems have been limited due to the need for large electrolyte reservoirs and liquid junctions. Disadvantages associated with the incorporation of liquid reservoirs in miniaturised devices includes fabrication defects, exhaustion of internal electrolyte and clogging of porous frits resulting in delayed responses and instability [18,19]. Thus it could be argued that the development of a miniature, reliable solid state reference electrode is equally important as the development of the nanoelectrode itself. The Ag/AgCl reference electrode is a widely used reference electrode because it is simple, inexpensive, very stable, non-toxic and its simplicity of fabrication and fundamental ruggedness makes it a good candidate for many industrial applications. Several authors have addressed the fabrication of miniaturised Ag/AgCl based references [20–24] for general use in micro electrochemical experiments with promising results. However, integration of a solid state reference electrode, concentric to a graphene micro ring working electrode whose electrochemically active surfaces can be replenished by attritive polishing, has to date, not been attempted.

Thus, the intent of this investigation is four fold: (1) to exploit the geometric and electrical properties of graphene to fabricate a robust microelectrode whose dimensions can be characterised and controlled; (2) to more efficiently exploit the greater electrochemical activity of graphene edge sites (as opposed to basal plane) in the electrode design; (3) to use said electrode to study the intrinsic electrochemical response of graphene by use of a well known redox probe and (4) to develop a minaturised reference electrode located concentrically to the microelectrode. To these ends we have fabricated the first Graphene Ring Micro Electrodes (GRiMEs) with integrated Ag/AgCl reference electrodes.

The advent of GRiMEs with concentric reference electrode capability opens up an opportunity for the fabrication of a durable, disposable and cost effective electrochemical sensor. The GRiME is therefore an excellent candidate for real-world analysis and suitable for portable electrochemical systems. Thus, the availability of easy-to-fabricate GRiMEs combined with a concentric Ag/AgCl reference electrode greatly expands the scope of both nanoelectrode and graphene-based electrochemical devices.

## 2. Experimental Section

All reagents used are AnalaR grade or higher, and purchased from Sigma Aldrich (Gillingham, Dorset, UK) or Alfa Aesar (Heysham, Lancashire, UK), and used as received. Fibre optics (220 μm UV/Vis, part # J59-300) were purchased from Edmund Optics, York UK. Clear epoxy casting resin/ hardener (Aeropia, Crawley, UK), SiC abrasive papers (Struers FEPA P#1000, Buehler-Met P1200, Struers FEPA P#2400), diamond polishing pastes (Marcon, Hitchin, UK) and aluminium oxide 0.015 μm (VWR, Leicester, UK) were used. Silver epoxy (2400 circuit works) was purchased from RS Components (Corby, UK). Distilled water was produced and further purified using a Direct Q UV3 Millipore system to a resistivity of 18.2 MΩ.cm (Millipore Limited, Watford, UK). Prior to electrochemical analysis, all reagent solutions were purged for 15 min with nitrogen (white spot grade, BOC Ltd: Lancaster, UK). Atomic force microscopy (AFM) was performed using a Veeco DI Calibre AFM operating in scanning mode. Unless stated otherwise, standard aluminium coated, silicon contact mode cantilevers with a tip radius of approximately 10 nm were used during AFM analysis purchased from Budget Sensors UK (Sofia, Bulgaria) Scanning electron microscopy (SEM) and energy dispersive X-ray (EDX) measurements were performed using an FEI Quanta 200 electron microscope with EDX attachment. Transmittance tests were carried out on a single wavelength spectrophotometer at λ = 550 nm (Model SP6-350, Pye-Unicam, Cambridge, UK). Raman analysis of the graphene coated slides was carried out using a Voyage 532 nm confocal Raman microscope. The power output of the 5 mW laser operated was operated at 25% power to prevent burning of the sample and a long integration time of 55 seconds. XRD analysis was carried out using a D2 Phaser XRD (Bruker, Coventry, UK).

Each individual cyclic voltammogram (CV) was recorded on a freshly cleaned electrode after pretreatment at 0 V for 5 seconds. All CV measurements were carried out using a three-electrode cell with a saturated calomel electrode (SCE) unless otherwise stated, and a Pt wire mesh (Advent, Oxford, UK) used as the reference and counter electrode respectively. The potentiostat used was an Autolab PGSTAT10 computer controlled workstation (Windsor Scientific Ltd., Slough, UK) equipped with an extreme low current detector (ECD). Electrochemical experiments were carried out in an earthed Faraday cage in order to minimise electrical noise and conducted at room temperature (298 ± 2 K). All cyclic voltammetric experiments were conducted in the absence of chloride containing electrolytes.

### 2.1. Graphite Oxide Preparation

The graphene used in this study was fabricated by chemical delamination of graphite by its oxidation to graphite oxide (GO). The GO generation was achieved by use of a solution phase, oxidative method [25] modified from the original Hummers method [26], a synthetic route that incorporates oxygen containing functionalities onto both the basal plane and edges of the component carbon layer structure of the graphite starting material. Briefly this method proceeds as follows: 5 g of carbon powder (<20 μm dia.) and 3.75 g of NaNO_3_ were added to 375 mL of conc. H_2_SO_4_. Using an ice bath to maintain a temperature of <293 K, 22.5 g of KMnO_4_ oxidising agent was then slowly added. After 5 days of constant stirring a brown GO suspension was formed. Impurities were removed from this suspension though 15 wash cycles. Each cycle included: centrifugation, removal of supernatant, addition 2 L of 3 wt% H_2_SO_4_/0.5 wt% H_2_O_2_ solution, stirring and sonication at 140 W for 30 min. Vacuum filtering of the remaining solution produced a dark brown filter cake of clean GO, which was dried under vacuum at 353 K and stored at room temperature in a sealed sample container. The dried GO was crushed to a course powder to aid dissolution.

Solutions of GO (0.1 and 10 wt%) were prepared in doubly deionised water (DDW) for substrate coating. Prepared solutions were subsequently sonicated to delaminate the GO and fully disperse the GO platelets within solution. The resultant GO suspension was then centrifuged at 1,000 rpm to remove any non-separated aggregates. The supernatant was separated from the centrifuged solid and reserved for the dip-coating stage. Supernatant GO concentrations were measured using thermogravimetric analysis (TGA, Shimadzu TGA-50, Milton Keynes, UK) under an inert N_2_ atmosphere. Final GO concentrations for 0.1 & 10 starting wt% solutions were found to be 0.07 (±0.009) & 3.24 (±0.29) wt% respectively. Inductively coupled mass spectrometry (ICP-MS) showed that the concentration of Mn ions remaining in solution was negligible at 3.76 ppb. Solutions were then stored until required for dip coating of substrates.

### 2.2. Quartz Slide and Fibre Optic Substrate Preparation

Two different substrates were prepared for coating with graphene; quartz slides for characterisation of the graphene material and fibre optics for GRiME fabrication. Slides were cleaned using 1:3 H_2_O_2_/H_2_SO_4_ for 1 h, rinsed in DDW and then dried in a N_2_ stream. They were then soaked in 3 wt% 3-(aminopropyl)triethoxysilane (APTES) in toluene for 1 h, creating an adhesion layer. Slides were removed from the mixture, rinsed in toluene, dried under a N_2_ stream and stored in a dessicator for use within 3 days. Coating of GO onto APTES-treated slides was carried out using an in-house dip coater. Slides were immersed into the 0.07 wt% GO solution at a rate of 44 μm/s, left suspended in solution for 1hr at room temperature before being withdrawn at a rate of 44 μm/s and left vertically suspended in the dip coater to dry. This produced faint brown layers of GO, visible only when placed over a white background. Dip coating covered both sides of the slide and therefore GO had to be removed from one side before optical transmittance measurements were made. This was done by wiping one side of the slide with a damp lens cloth. Slides were then stored in a dessicator prior to GO reduction.

Fibre optics (110 μm radius) were prepared using fibre lengths cut to 60 mm. These were washed in 1:3 H_2_O_2_/H_2_SO_4_ for 1 h, rinsed with DDW and dried under a N_2_ stream. The fibres were then silanised to improve adhesion [27]. By refluxing for 10 min in a mixture of 50.8 mL propan-2-ol, 1 mL APTES and 1 mL DDW followed by rinsing in propan-2-ol and drying at 423K for 3 min. This process was then repeated once. Treated fibres were stored in a dessicator until dip coated (use within 3 days). Fibres were lowered into a 3.24 wt% GO solution at a rate of 85 ± 1 mm/s and instantly retracted at the same rate leaving a light brown adhered layer. They were then left to dry at room temperature inside a dessicator. Thicker layers were obtained by repeating the process at 1 min intervals. Prepared fibre optics were stored in a dessicator prior to GO reduction.

### 2.3. Reduction of GO Layers to Graphene

The so-formed layers of GO were reduced to graphene by a two stage process. The first step involved the exposure of the GO coated slides and fibre optics to fuming hydrazine monohydrate at 313 K for 20 h forming hydrazinium graphene (HG). For the second step, HG coated slides or fibre optics were thermally annealed in an inert N_2_ atmosphere. In order to achieve this, a furnace was heated to 973 K at a rate of 300 K/h and this temperature was held for 1 h. A constant inlet N_2_ flow of 5 L/min was maintained throughout. The furnace was then allowed to cool to room temperature before isolating the N_2_ supply and removing the sample. The slide prepared graphene layers were characterised by SEM, energy dispersive *x*-ray analysis (EDX), AFM and UV-visible spectroscopy (transmittance values).

### 2.4. Reference Electrode Preparation

To fabricate the Ag/AgCl reference electrode, glass capillary tubes were first metallised through photocatalytically initiated electroless deposition (PIED) [28], a novel method for the deposition of metal onto insulating substrates using a semiconductor photocatalyst previously developed in our laboratory.

#### 2.4.1. TiO_2_ Sensitisation by Sol-Gel

Glass capillary tubes were sensitised with a mesoporous TiO_2_ layer via sol-gel dip coating. A reversed micellular sol-gel, as described by Yu *et al.* [29], is first prepared by firstly mixing Triton X-100 (26 g) and cyclohexane (150 mL). After 30 mins, water (1.08 g) is added and the solution becomes turbid. This turbidity clears upon addition of titanium isopropoxide (23 g) after which acetylacetone (10 mL) is added to stabilise the solution. The resultant sol-gel was then applied to a glass capillary tube via dip coating at a rate of 1 mm/s using the apparatus described in Section 2.2. Coated capillary tubes were then fired in a furnace at 773 K for 1 h to anneal the TiO_2_ and produce a resilient coating. Annealing at this temperature produces TiO_2_ with predominantly anatase structure [28]. Sensitised substrates were stored in the dark at room temperature before use to ensure no absorption of UV light occurred.

#### 2.4.2. Preparation of Ag Electroless Plating Solution

A silver electroless plating solution was prepared with a composition as follows: Silver Nitrate 1.49 g/dm^3^; ethylenediamine 3.24 g/dm^3^; potassium sodium tartrate 0.73 g/dm^3^; 3,5-diiodotyrosine 1.70 g/dm^3^. These components are added to a small quantity of distilled water in the order listed, ensuring full dissolution with each addition. The completed solution is made up to volume with distilled water at a temperature of 298 K. Electroless plating solutions are freshly made immediately before use for optimum performance. As silver nitrate is light sensitive silver solutions are prepared and stored in amber flasks.

#### 2.4.3. One-Step Photocatalytically Initiated Electroless Deposition (PIED)

**Figure 1.**
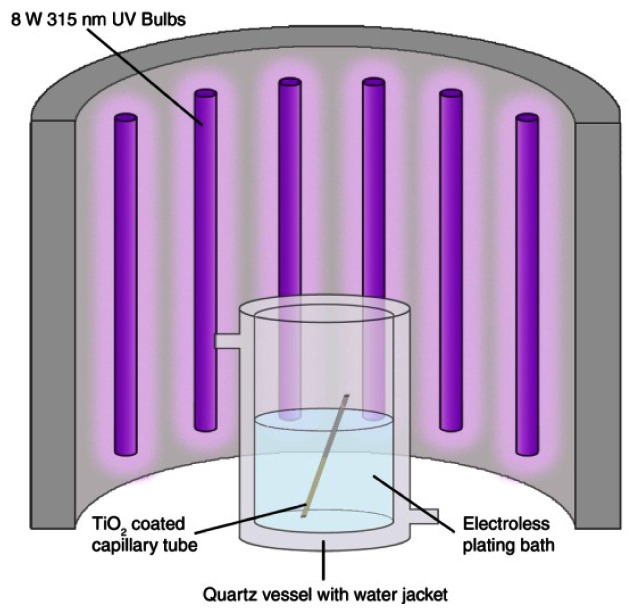
PIED Experimental Setup.

TiO_2_ sensitised substrates (prepared as in Section 2.4.1) are placed directly into freshly prepared electroless plating solutions in a quartz reaction vessel (Figure 1). Quartz vessels are used for improved UV transmittance in comparison to glass. An N_2_ stream is applied and bubbled through the plating solutions for the remainder of the experiment in order to purge the solution of oxygen, so preventing O_2_ competing with metal ions for reduction at the photocatalytic surface. The N_2_ bubbles also provide a source of agitation to prevent local depletion of metal ions at the substrate surface. The reaction vessel is then placed inside an enclosed UV light-box and irradiated with 12 × 8 W UV A lamps (Figure 1) for 1 h after which time a homogeneous silver layer is produced across the TiO_2_ sensitised substrate surface.

#### 2.4.4. Chlorination of Ag Plated Glass Capillaries

Silver plated capillaries were converted to AgCl by immersion in saturated KCl for 30 min at 298 K under a blanket of N_2_ nitrogen in the dark in order to avoid re-oxidation to metallic Ag and Cl^−^ after which, silver chloride layers took on a pale purple hue. Post chlorination, electrical contact was made to the AgCl layer using silver loaded epoxy resin, thus establishing the Ag/AgCl boundary. It should be noted that typical manufactured reference electrodes are placed into an aqueous solution containing a known concentration of chloride ions so that a potential determining equilibrium can be established at the Ag/AgCl boundary. In this case the formed Ag/AgCl ring electrode was not placed into chloride containing solution, with only chloride ions in the silver chloride layer being used to maintain equilibrium. Therefore in order to use so-formed Ag/AgCl reference electrodes during cyclic voltammetric experiments it is imperative that electrolytes containing no chloride ions are used so as to avoid any potential drift of the reference potential.

### 2.5. GRiME/Ag/AgCl Electrode Construction & Tip Preparation

**Figure 2.**
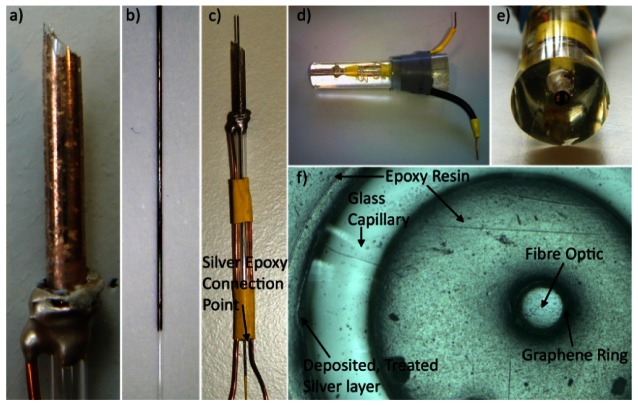
Stages of fabrication of the GRiME and concentric Ag/AgCl reference electrode. (**a**) Bare copper wire attached to the Ag/AgCl layer present on the capillary tube using Ag loaded epoxy. (**b**) Un-potted GRiME. (**c**) Un-potted assembly comprised of the Ag/AgCl reference, the GRiME and relevant wire connections. (**d**) Side view of the completed assembly. (**e**) End view of the completed assembly. (**f**) Optical microscopy image of the tip of the GRiME and Ag/AgCl reference electrode.

Using silver-loaded conducting epoxy, a bare copper wire was attached to the Ag/AgCl layer present on the capillary tube and left to harden (Figure 2(a)). Heat-shrink tubing was used to secure both the copper wire coming from the Ag/AgCl layer to the capillary wall and an additional wire to the side of the capillary for making connection to the electrode material present on the fibre optic. Next, the centre of the capillary tube was filled with epoxy resin using a pipette and the GRiME (Figure 2(b)) was positioned down the centre of the capillary tube, allowing two days to harden. Silver-loaded conductive epoxy is then used to make a connection between the graphene layer and second copper wire (Figure 2(c)). The resultant capillary/fibre optic assemblies were then cast in clear epoxy resin using a large pipette tip as a mould. After the epoxy had hardened the end tip of the mould and associated cast epoxy were cleaved with a fine toothed saw exposing the ring electrode tips. The whole assembly was then de-shelled from the mould (Figure 2(d,e)). The electrode tip was polished using decreasing grades of SiC abrasive papers, followed by decreasing grades of diamond slurries (particle sizes: 6, 3, 1 μm), an alumina slurry (particle size: 0.015 μm) and a final polish on a clean polishing pad soaked in distilled water. After polishing the electrode tip was sonicated for 15 seconds at 20 W power in ethanol followed by distilled water to remove any debris remaining from the polishing stages producing a tip which was optically transparent and free from polishing debris, Figure 2(f).

## 3. Results and Discussion

### 3.1. GO and Reduced GO; Material Characterisation

**Figure 3.**
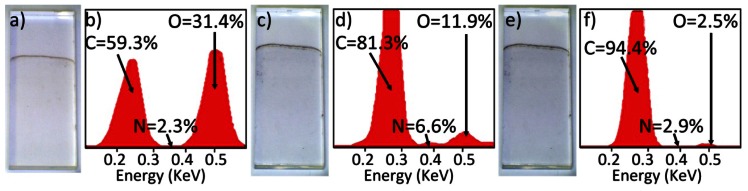
(**a**) GO layer. (**b**) GO EDX analysis. (**c**) Hydrazine reduced HG layer. (**d**) HG EDX analysis. (**e**) Hydrazine and thermally reduced, graphene layer. (**f**) Graphene EDX analysis.

Figure 3(a,b) show a faint brown, as deposited GO layer on quartz and its associated EDX analysis respectively. Following the first stage of reduction via hydrazine fuming to form HG, this layer becomes grey in appearance (Figure 3(c)), associated EDX in Figure 3(d). It is evident from the EDX analyses that hydrazine reduction partially removes some oxygen content from the GO material, and incorporates nitrogen containing functionalities within the HG. After the second stage of reduction via thermal treatment, HG is converted to graphene and the layer displays a glossy black aesthetic, Figure 3(e). The associated EDX indicates a near complete removal of oxygen content (Figure 3(f)).

The average transmittance of light at a wavelength of 550 nm of the GO layers, calculated from measurements made on 8 similarly prepared slides, was 99.1 ± 0.3%. The layers were found to have a surface resistivity exceeding 20 MΩ. In contrast, the graphene layers of Figure 3(e) had a surface resistivity of 27.7 KΩ/sq and a light transmittance of 87.8% at 550 nm. These values are comparable to, if not better than, those reported in work by Becceril *et al.*[25], whereby a spin coated GO layer was prepared onto quartz slides and reduced by hydrazine vapour fuming followed by annealing in argon at 673 K, producing a surface resistivity of 31 KΩ/sq and a light transmittance of 78% at 550 nm. Additionally, the light transmittance of 87.8% for the layer shown in Figure 3(e) is consistent with the films being comprised of an average of 5.3 graphene layers, assuming the widely accepted absorbance value of 2.3% per single graphene layer [30].

**Figure 4.**
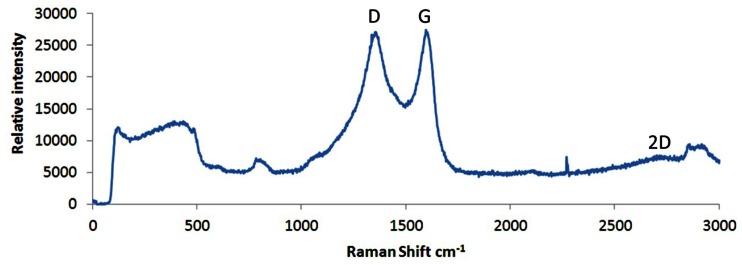
Raman spectra of graphene prepared on a quartz substrate.

Figure 4 shows the Raman spectra of the layer shown in Figure 3(e,f) (above) of the reduced/annealed films produced in this work. This is consistent with the Raman spectra recorded by Stankovich *et al.* [31] and, more recently, Willemse *et al.* [32] of chemically delaminated, defected graphene, produced from graphite oxide as a precursor. Present within the spectra of Figure 4 is both a G band at 1,582 cm^−1^ and a D band at 1,350 cm^−1^. The former is derived from the E_2g_ in-plane vibrational mode within aromatic rings and thus is indicative of the degree of graphitisation [33]; the latter relates to sp^3^ carbons in the presence of isolated domains of aromaticity and so is indicative of the number of graphene flake edge sites or residual sp^3^ sites associated with unreduced epoxide/hydroxyl groups [34]. The D band is a defect induced Raman feature and would not be seen in a purely crystalline sample [35]. An additional peak attributed to the 2D band is usually observed in graphene samples occurring at a shift of ∼2,685 cm^−1^ and is second order to the D band. This band is the result of lattice vibrational processes but unlike the D band, it does not need to be activated by proximity to a defect and is very sensitive to graphene folding [36]. The 2D band present within the spectra shown in Figure 4 is not as strong as would be expected as it is usually very strong within a pure, defect free graphene sample, even when no D band is present. A peak is present at ∼2,850 cm^−1^ which could be attributed to a wavelength induced shift of a multilayered material [36]. The wide peaks occuring below 900 cm^−1^ are derived from the underlying quartz substrate.

**Figure 5.**
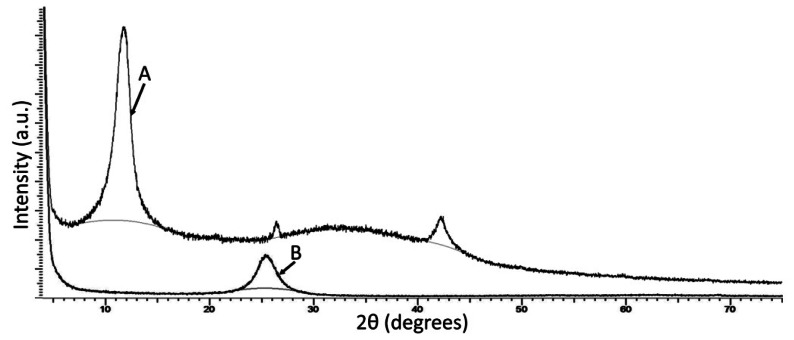
XRD spectra of (A) as prepared GO and (B) reduced GO/graphene.

Additionally, we also characterised the graphene layers used in this work by XRD analysis. In this analysis, our GO samples show a very strong peak at 11.2°, whilst the reduced GO/graphene samples exhibit their strongest peak at 25.5°, Figure 5. These figures are in good agreement with the results of Willemse *et al.*, who report peak shifts for GO and graphene of 9.8° and 24.88° respectively.

**Figure 6.**
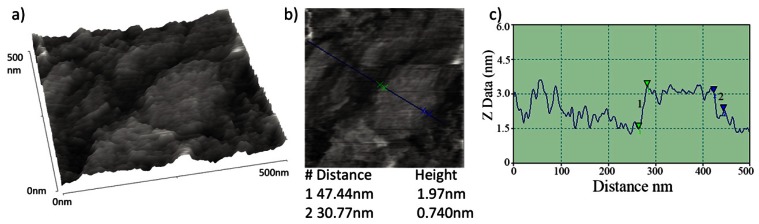
(**a**) Topographical contact mode AFM image of the graphene layer shown in Figure 3(e). (**b**) 2D, aerial view, of the same area. (**c**) Profile analysis of same, showing step heights of 1.97 and 0.74 nm, for arrow sets 1 and 2 respectively.

Topographic characterisation of the graphene layers on quartz slides was carried out using AFM. Contact mode images of the graphene layer of Figure 3(e) are shown in Figure 6. These AFM images exhibit some variability in the height of the flakes comprising the graphene layer. Z direction data within Figure 6(c) shows minimum sharp height changes that are consistent with the presence of some bilayer graphene flakes within a broadly multilayer sample, the multilayer flakes forming a coherent conductive layer. Such height changes are similar in both sharpness and magnitude to those observed from isolated graphene platelets produced by an analogous route by Stankovich *et al.* [31].

**Figure 7.**
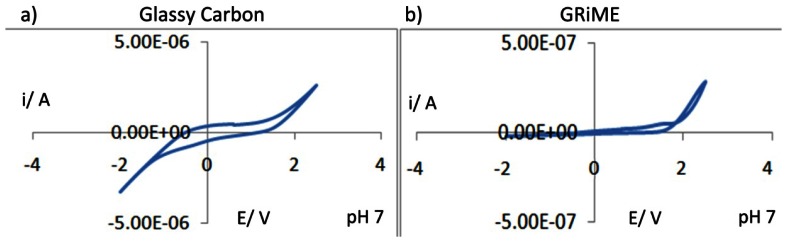
Comparison of CV's recorded in aqueous background at pH 7 for a glassy carbon disk electrode (4 mm dia.) and a GRiME. CV's were recorded over a potential range from −2 to 2.5 V at a scan rate of 150 mV/s *vs.* SCE.

Electrochemical characterisation of the GRiMEs was first carried out by performing cyclic voltammetric experiments in pH 7 aqueous background solutions within a 4.5 V potential window. For purposes of comparison, a CV of glassy carbon is presented under similar conditions. CV's recorded at the GRiME and at glassy carbon were found to be similar supporting our conclusion that the GRiMEs are comprised of a carbon based electrode material *i.e.*, multilayer graphene. Furthermore the GRiMEs exhibit a wider water solvent window than glassy carbon, Figure 7(a,b).

It was expected that any remaining oxygen functionalities existing within the graphene comprising the GRiME (for example 1,2- and 1,4-quinone structures residing at graphene flake edges [37–39]), would result in peaks assosciated with their reduction/ re-oxidation in the CVs shown in Figure 7. However, such peaks were not observed on CVs recorded within a potential window ranging from −2 to 2.5 V suggesting the absence of such oxygen-containing groups, further supporting our conclusion that the samples of Figures 3(e,f), 4 and 5(b) are predominantly graphene. Based on the CVs of Figure 7(b) a potential window ranging from −0.45 to 1.0 V was selected for further voltammetric experiments.

**Figure 8.**
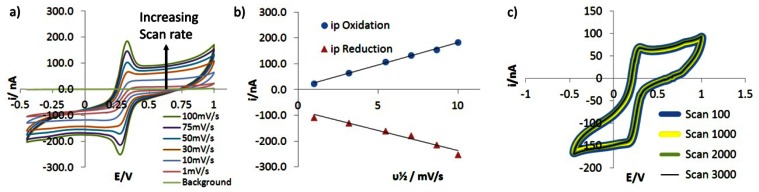
(**a**) CVs recorded at a GRiME at a range of sweep rates in a solution of 5 mmol/L Fe(CN)_6_^3−^ and 1 mmol/L KCl_,_ pH 2. The starting potential was +0.3 V *vs.* SCE scanning in the positive direction at onset. The solution was deoxygenated using an N_2_ stream for 10 min prior to data acquisition. (**b**) anodic and cathodic peak currents of Figure 8(a) plotted as a function of the square root of the scan rate. (**c**) 3,000 scan overlay recorded at 500mV/s.

The GRiMEs were then tested in a solution containing 5 mmol/L Fe(CN)_6_^3−^, 1 mol/L KCl at pH 2, over a range of potential sweep rates from 1–100 mV/s, the results of this analysis are shown in Figure 8.

Figure 8(a) shows a simple, sigmoidal pseudo-steady state voltammogram is observed at a sweep rate of 1 mV/s. No evidence of any uncompensated resistance is seen in this CV indicating that, due to the nA scale currents passed through the micro-ring, the intrinsic resistance of the graphene electrode (expected on the basis of the sheet resistance reported above for similarly prepared graphene coated slides) is having no effect on the electrochemical current-voltage response.

As the scan rate increases above 30 mV/s small, peaked current responses at 0.25 and 0.32 V are observed in the CV of Figure 8(a). This is due to the increase in the rate of electron tranfer, with increasing potential outstripping the rate at which the concentration profile within the 220 μm diameter microring elecrode's hemispherical diffusion field adjusts through action of mass transfer to reflect that rate of electron transfer. This is despite the efficiency of diffusive mass transport at microring electrodes, indicating an intrinsically high rate of interfacial electron transfer to ferricyanide from the graphene edge sites that comprise the electroactive surface of the GRiME. Such rapid electron transfer would be expected to lead to a reversible electrochemical response from the system. This is confirmed by the separation of the cathodic and anodic peak potentials being near invariant with an average value of 55 mV compared with the expected 59.1 mV [40] for a geniune reversible process, Figure 8(a); and by the cathodic and anodic peak currents both showing a simple linear dependence on the square root of the sweep rate, Figure 8(b). The latter conformity with Randles-Sevich behaviour suggests that electron transfer is occcurring between the GRiME and a free solution species rather than a surface adsorbed entity or within a porous electrode structure. Further, this system was cycled for >3,000 scans and the voltammograms were found to overlay without deviation as shown in Figure 8(c) indicating that the electrochemical response of the GRiME is highly stable and reproducible.

### 3.2. Sizing of the GRiMEs

Electrochemical sizing of the GRiME was carried out using existing mathematical models of microring behaviour. Equation 1 describes the steady state diffusion limited currents (*i_diff,lim_*) obtained at microring electrodes with inner to outer ring radii ratios of greater than 0.99 [41]:

(1) $$i_{\text{diff},\lim}={\text{nFDCl}}_{0}$$

where *n* is the number of electrons transferred, *F* the Faraday constant, *D* the diffusion coefficient (m^2^/s), and *C* is the concentration of electroactive species (mol/L). By recording chrono-amperograms with the GRiMEs under conditions of diffusion control and plotting the observed, diffusion controlled steady state currents as a function of [Fe(CN)_6_^3−^] in accordance with Equation (1), a straight line with a slope equal to *nFDl_0_* may be obtained. The parameter *l_0_* is related to the inner, *a*, and outer, *b*, ring radii through Equation (2) [42]. The inner radius *a* is known (110 μm), allowing for simple evaluation of *b* and thus the ring thickness *b*-*a* from *l_0_*:

(2) $$l_{0}=\frac{\pi^{2}(a+b)}{\text{In}\left[16\left(\frac{b+a}{b-a}\right)\right]}$$

To this end chronoamperograms were recorded at two different graphene thickness GRiMEs over a range of [Fe(CN)_6_^3−^] at pH 2, adjusted using HNO_3_, and steady state currents obtained. For these measurements, the GRiME working potential was stepped to −0.1 V *vs.* SCE as, from Figure 8(a), a stable, mass transport controlled faradaic response is observed at this potential. From a plot of *i_diff, lim_ vs.* [Fe(CN)_6_^3−^], an average value of *l_o_* of 2.016 × 10^−4^ m ± 1.785 × 10^−5^ m and 1.763 × 10^−4^ m ± 1.386 × 10^−7^ m was obtained for GRiMEs fabricated using four dip and three dip coatings of GO respectively. From Equation 2, and for *a* = 110 μm, this value of *l_o_* equates to ring thicknesses of 73.8 nm and 15.8 nm, respectively, both therefore having an *a*/*b* ratio > 0.999, thus validating the use of Equation (1). The latter three dip coat electrode was used to fabricate a GRiME with a concentric Ag/AgCl reference electrode.

### 3.3. Deposited TiO_2_ and Ag: Material Characterisation

**Figure 9.**
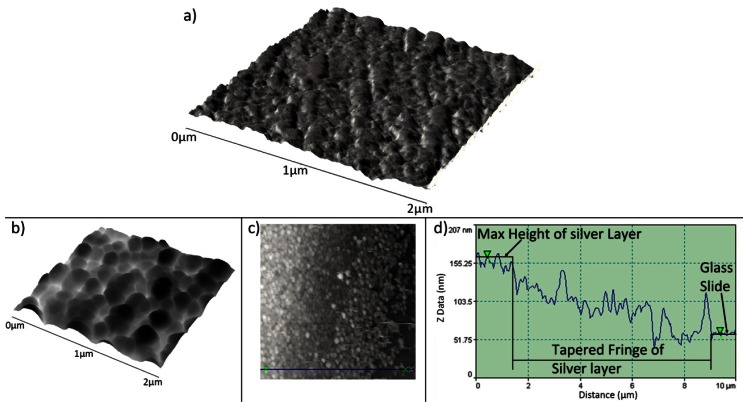
(**a**) Mesoporous structure of anatase TiO_2_ layer. (**b**) AFM topography of the silver metal grains within a silver layer deposited on a TiO_2_ sensitised microscope slide. (**c**) AFM image of the silver layer/glass slide fringe. (**d**) AFM topography step height measurement over a distance of 9.10 μm and a height change of 104.8 nm.

TiO_2_ sol-gel coated capillary tubes were fired in a furnace at 773 K for 1 h to anneal the TiO_2_ and produce a resilient coating. This process was also carried out on a TiO_2_ coated quartz slide enabling investigation using AFM. Annealing at this temperature produces TiO_2_ with predominantly anatase structure [28] and coatings are composed of interconnected, mono-dispersed, spherical primary particles within a mesoporous structure, Figure 9(a). The TiO_2_ sensitised substrates were then treated in an electroless plating solution as described in Section 2.4.3. Upon completion the substrate is removed from the plating solution, rinsed with distilled water and dried in air. Analysis of the silver layer produced using the above method was carried out on a plated flat microscope slide using an AFM operating in scanning mode, Figure 9(b). An average TiO_2_/silver layer thickness of 118.35 ± 15.00 nm was obtained by measuring the step height of 8 different areas at the layer/substrate fringe, Figure 9(c,d). After treatment of the so-formed silver layer in saturated KCl (as described in Section 2.4.3) the layer took on a pale purple hue after being converted to AgCl, as confirmed using EDX analysis (not shown). An example of this layer can be seen in Figure 2(a).

### 3.4. Electrochemical Performance of the Concentric Ag/AgCl Reference Electrode

In aqueous solution a potential difference of 45mV exists between a saturated KCl, Ag/AgCl reference electrode and a saturated KCl, SCE reference electrode [30]. Thus, in order to determine the shift in potential existing between the GRiMEs integrated concentric Ag/AgCl reference electrode and a saturated SCE, both references were placed into a beaker containing a model aqueous solution of 5 mmol/L Fe(CN)_6_^3−^ and 1 mol/L KNO_3_ at pH 2 adjusted using HNO_3_. Using a digital volt meter (DVM), 12 separate potential measurements were taken over a period of 2 weeks between the concentric Ag/AgCl and SCE reference electrodes. Between each measurement, the concentric Ag/AgCl electrode was polished (as described in Section 2.5) to expose a new region. From a total of 12 measurements a potential of 41.0 ±3.63 mV was obtained for the concentric Ag/AgCl reference *vs.* SCE. This is in good agreement with the standard potential difference. The concentric Ag/AgCl reference was also tested in the same way after 2 days without polishing. An average potential difference *vs.* SCE of 36.15 ± 2.50 mV was observed revealing that a drop ∼5 mV had occurred from previous measurements. This potential difference is potentially due to the formation of a silver oxide layer at the exposed surface of the reference electrode. Therefore the electrode was removed from solution, polished as per the polishing steps described in Section 2.5 and re-tested in the same way. This time an average potential difference of 43.33 ± 2.50 mV was recorded, thus illustrating that it is imperative that the concentric Ag/AgCl reference is cleaned by attritive polishing prior to use. However, as attritive polishing is facilitated within the design of the GRiME this is of no detriment to the use of the sensor and a new surface can be exposed should electrode poisoning occur.

**Figure 10.**
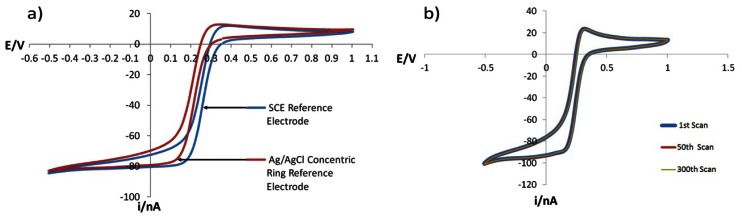
(**a**) CV's recorded at the GRiME at a scan rates of 50 mV/s. The starting potential was +0.35 V with respect to each reference electrode and the initial scan was in the direction of increasing positive potential. (**b**) 1st, 50th and 300th scan overlay CV recorded at 250 mV/s in the same solution.

A CV experiment was conducted again using a solution of 5mmol/L Fe(CN)_6_^3−^ and 1 mol/L KNO_3_ at pH 2 adjusted using HNO_3_ and using the GRiME as the working electrode to further assess the performance of the concentric Ag/AgCl reference *vs.* SCE. From Figure 10(a) it can be seen that there is a 41 mV difference between anodic/cathodic peak potential separation when the data is overlaid, which is again in agreement with the data presented by Bard and Faulkner [40] as well as the DVM measurements described above.

In order to further assess the of the concentric Ag/AgCl reference electrode within the context of the GRiME sensing system a similar experiment as above was conducted over 300 scans. The scans were overlaid, and as can be seen in Figure 10(b), the traces overlay without deviation thus demonstrating the stability and therefore the reliability of the GRiME and integrated reference when operating at nA scale currents.

## 4. Conclusions

For the first time we have successfully fabricated a graphene ring microelectrode (GRiME) as a working electrode with a concentric Ag/AgCl reference electrode. This was made possible by one-step photocatalytically initiated electroless deposition (PIED) of silver layers onto glass capillary tubes with addition of a GRiME working electrode in the centre of the capillary tube.

GRiMEs were fabricated by dip coating chemically delaminated graphite oxide followed by a simple two step reduction process leading to the production of conducting nano layers based on a graphene material. The resultant GRiMEs had ring electrode thicknesses of ∼73 and 15 nm prepared by dip coating four and three times, respectively, corresponding to inner to outer ring ratios in excess of 0.99. Electrochemical characterisation of the device using Fe(CN)_6_^3−^ has shown highly reversible, stable (response invariant over >3,000 scans) and reproducible electrochemistry. The microring design allows for efficient use of electrochemically active graphene edge sites and the nA scale currents associated with GRiMEs neatly obviate issues relating to the high resistivity of undoped graphene. Additionally we have demonstrated the use of a minaturised Ag/AgCl reference electrode, concentric to the GRiME working electrode. The reference electrode was shown to exhibit high stability (response invariant over >300 scans) indicating that the inherent design of the GRiME and integrated reference electrode is highly reliable when operating at nA scale currents. This study demonstrates the potential of the GRiME for the investigation of electrochemical systems, either industrial or research based, possibly finding use in process control, environmental monitoring or for *in vivo* studies.

Future work will focus on the accessing of electrode thicknesses closer to the 0.334 nm layer thickness of graphene, so allowing for nanoelectrochemical studies of fundamental processes using electrodes with sub-nm dimensions for the first time. Additionally, the use of GRiMEs to study photoactive species will be investigated, utilizing the central fibre optic as a light guide.
